# Beyond Coding Variants: RNA-Level Mechanisms in Human Disease and Precision Therapeutics

**DOI:** 10.3390/genes17070777

**Published:** 2026-06-30

**Authors:** Himanshu Goel

**Affiliations:** 1 Hunter Genetics, P.O. Box 84, Waratah, Newcastle, NSW 2298, Australia; himanshu.goel@health.nsw.gov.au; Tel.: +61-2-49853100; Fax: +61-2-49853105; 2 School of Medicine and Public Health, University of Newcastle, Callaghan, Newcastle, NSW 2308, Australia

**Keywords:** RNA processing, splicing, nonsense-mediated decay, RNA surveillance, RNA-binding proteins, non-coding RNA, translational regulation, upstream open reading frames, synonymous variants, RNA therapeutics, precision medicine, rare disease

## Abstract

Clinical genomics has traditionally focused on protein-coding variation, yet many pathogenic mechanisms arise through alterations in RNA processing, stability, localisation, translation, and surveillance. Prior reviews have addressed individual RNA layers, splicing, non-coding RNAs, RNA therapeutics, or RNA diagnostics in isolation. This review presents an integrated, mechanism-matched framework linking RNA-level disease mechanisms to diagnostic reasoning and therapeutic selection across all major RNA layers, offering a practical resource for clinical geneticists and translational researchers. I examine how splicing defects, pseudoexon inclusion, polyadenylation disruption, RNA editing loss, untranslated-region variants, premature termination codons, stop-loss variants, RNA-binding protein dysfunction, non-coding RNA dysregulation, altered codon usage, ribosome stalling, and surveillance pathway failure, including nonsense-mediated decay, nonstop decay, and no-go decay, each create distinct and mechanistically addressable disease states. A central argument of this review is that treatment selection must be mechanism-matched rather than gene- or variant-class-based: splice defects may require antisense oligonucleotide (ASO)-mediated correction or small-molecule splice modulation; toxic transcripts may require ASO- or siRNA-mediated silencing; haploinsufficiency may require mRNA replacement or transcript rescue; premature termination codons are candidates for readthrough only when transcript and protein context are favourable. I further argue that RNA sequencing, long-read transcriptomics, allele-specific expression analysis, and functional assays are essential for both diagnosis and therapeutic stratification. The framework described here moves clinical variant interpretation beyond descriptive classification toward mechanism-based, RNA-centric precision medicine.

## 1. Introduction

**Figure 1:**
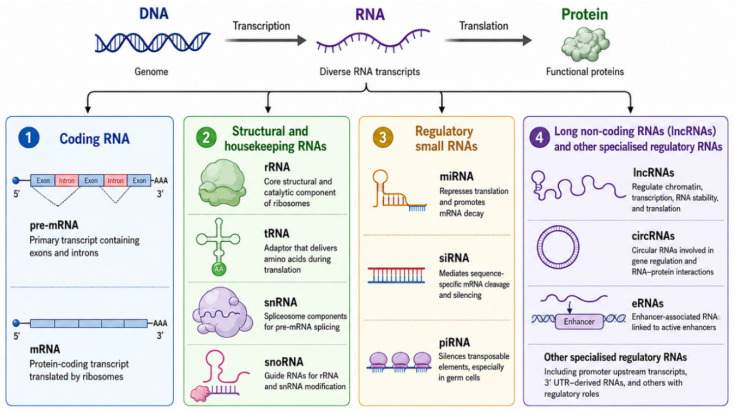
Major Types of RNA in Human Cells.

RNA is increasingly recognised as a central regulatory layer in cellular biology rather than a passive intermediary between DNA and protein. Advances in transcriptomics and RNA biology have demonstrated that aberrations at the RNA level, including defects in splicing, RNA stability, non-coding RNA regulation, and RNA editing, play a central role in the pathogenesis of diverse human diseases, including inherited disorders, cancer, and neurodegenerative conditions [1,2]. MicroRNAs and other small regulatory RNAs fine-tune transcript abundance and translational output [3,4]; long non-coding RNAs regulate chromatin organisation, transcription, promoter and enhancer activity, RNA stability, and RNA–protein interactions [3,4,5]. Together, these RNA species form a major regulatory layer relevant to cellular function, disease, and therapy, as illustrated in Figure 1.

DNA is transcribed into diverse RNA species, only some of which are protein-coding. Structural, small regulatory, and long non-coding RNAs coordinate translation, splicing, gene silencing, and gene regulation, forming a key regulatory layer in cellular function, disease, and therapy. This figure illustrates selected examples of functionally important RNA classes; the full repertoire of cellular RNA species is considerably larger.

The concept of “beyond coding variants” encompasses two related concepts. First, pathogenic variants may occur outside the protein-coding sequence, including splice sites, branch points, intronic regulatory elements, untranslated regions, polyadenylation signals, non-coding RNA genes, and distal regulatory elements [3,6,7,8]. Second, variants within coding sequence may exert their primary pathogenic effect not through amino acid substitution but through disruption of RNA-level information rather than alteration of the amino acid sequence. Exons simultaneously encode proteins and contain splicing regulatory elements, RNA-binding protein recognition motifs, translational control signals, and determinants of RNA stability [9,10]. As a result, variants with similar genomic classifications may produce markedly different biological consequences. A nonsense variant may trigger nonsense-mediated decay and cause haploinsufficiency [8], or escape RNA surveillance and produce a dominant-negative protein [11]. A synonymous variant may disrupt splicing despite preserving amino acid sequence, whereas a deep intronic variant may activate a pseudoexon and introduce a premature termination codon [9,10]. Pathogenicity is therefore often determined by transcript consequence rather than variant class alone.

These principles have important implications for genomic diagnosis. Although exome sequencing remains the foundation of rare disease diagnostics, many pathogenic variants act through RNA mechanisms that are poorly captured by coding-centric analytical frameworks [12,13]. Deep intronic splice-altering variants, untranslated-region variants affecting transcript regulation, synonymous variants disrupting splicing, and repeat expansions producing toxic RNA species may all be underdetected or misinterpreted by conventional approaches [14,15]. RNA sequencing and related transcriptomic technologies address this limitation by directly interrogating transcript structure, abundance, and function, and have been shown to improve diagnostic yield in patients who remain unsolved following exome or genome sequencing [16]. The growing ability to define RNA-level disease mechanisms has also transformed therapeutic development. RNA is inherently accessible to sequence-specific intervention, enabling therapeutic modulation of transcript abundance, splicing, translation, and sequence composition. Current RNA-targeted strategies include antisense oligonucleotides, small interfering RNAs, messenger RNA replacement therapies, splice-modulating compounds, and programmable RNA-editing technologies. Clinical success across disorders such as spinal muscular atrophy (SMA) [13,16], transthyretin amyloidosis [17], acute hepatic porphyria [18,19], and SOD1-associated amyotrophic lateral sclerosis [20,21] has established RNA-directed intervention as a practical therapeutic modality rather than a purely experimental concept.

Recent reviews have examined individual aspects of RNA biology in isolation: splicing mechanisms and their disease consequences [1,2], non-coding RNA regulation [3,4,5], transcriptomic diagnostic approaches [22,23] and RNA-targeted therapeutic platforms [24]. None of these works systematically connects all three domains, variant class, diagnostic approach, and therapeutic selection, within a single integrated framework. The critical clinical gap is that mechanistic understanding of an RNA defect and knowledge of available RNA therapeutics are rarely synthesised into actionable guidance: a clinician identifying a pseudoexon-generating deep intronic variant needs to know simultaneously how to confirm it transcriptomically, why it is not a readthrough candidate, and why a steric-blocking ASO is the appropriate intervention. This review provides that integrated, mechanism-matched framework, linking each class of RNA-level variant consequence to the diagnostic assay needed to detect it and the therapeutic strategy best suited to correct it. This synthesis constitutes its specific contribution beyond the existing literature.

Literature selection for this review was performed by searching PubMed and Google Scholar using terms including RNA processing, splicing variants, NMD, RNA-binding proteins, non-coding RNA, translational regulation, RNA therapeutics, antisense oligonucleotides, and related clinical disease terms. Priority was given to primary research articles, systematic reviews, and pivotal clinical reports published between 2015 and 2026, supplemented by landmark earlier studies considered foundational to each mechanism. No formal systematic review methodology or PRISMA protocol was applied; the selection reflects the author’s judgment of representative, high-impact, and clinically relevant studies.

**Figure 2:**
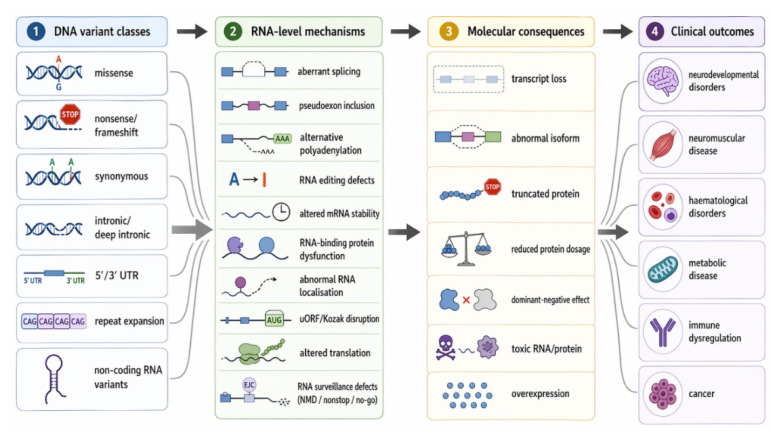
RNA as the Interpretive Layer between Genotype and Phenotype.

The review is organised into five major sections. Section 2 examines RNA-level mechanisms of disease. Section 3 discusses diagnostic approaches for detecting RNA abnormalities. Section 4 reviews therapeutic strategies matched to specific RNA mechanisms. Section 5 evaluates the clinical maturity, translational limitations, and regulatory challenges associated with RNA-targeted therapies. Section 6 explores future directions, including multi-omic integration, artificial intelligence-assisted interpretation, and increasingly personalised RNA medicines. Together, these sections provide a clinically oriented framework linking molecular mechanisms, diagnosis, and therapeutic stratification in RNA-guided precision medicine. The relationship between variant class, RNA-level consequence, and downstream molecular outcome is summarised in Figure 2.

DNA variants shape phenotype through RNA-level effects on splicing, stability, localisation, translation, and surveillance. Diverse variant classes converge on core mechanisms, aberrant splicing, dosage imbalance, and translational disruption, resulting in loss of function, toxic species, or altered protein output across human disease.

## 2. RNA-Level Mechanisms of Disease

### 2.1. 5′ Capping and Cap Recognition, and Pre-mRNA Splicing

The 5′ cap is added soon after transcription begins and supports mRNA stability, nuclear export, and translation initiation. Although primary disorders of core capping enzymes are not a common Mendelian disease category, disruption of cap-dependent translation is clinically relevant in neurodevelopment. Decapping scavenger enzyme (DCPS) removes residual 5′ cap structures generated during mRNA degradation. Biallelic loss-of-function variants in the *DCPS* cause a rare autosomal recessive neurodevelopmental disorder characterised by developmental delay, intellectual disability, craniofacial dysmorphism, and neuromuscular abnormalities, with functional studies demonstrating complete loss of decapping activity and accumulation of abnormal capped RNA metabolites [25,26].

### 2.2. Pre-mRNA Splicing

Disruption of pre-mRNA splicing is one of the most important RNA-level disease mechanisms. Accurate exon recognition depends on coordinated interactions between conserved splice donor and acceptor sites, branch points, polypyrimidine tracts, and auxiliary exonic and intronic splicing regulatory elements. Although canonical splice-site variants are well recognised causes of disease, pathogenic splicing defects may also arise from synonymous variants, exonic missense variants, deep intronic changes, branch-point variants, and alterations affecting splicing enhancers or silencers [9,10].

Splicing abnormalities can result in exon skipping, intron retention, cryptic splice-site activation, or pseudoexon inclusion. These events may alter the reading frame, introduce premature termination codons, trigger nonsense-mediated decay (NMD), or generate abnormal protein isoforms with altered function [11]. Consequently, the pathogenic impact of a variant is often determined by its effect on transcript architecture rather than by its predicted protein consequence alone. Alternative splicing further expands transcript diversity and is particularly important in the nervous system, where tissue-specific isoforms contribute to neuronal development, synaptic specification, and circuit formation. Genes such as *NRXN1* and *DSCAM* generate extensive isoform repertoires that influence neuronal connectivity and signalling [27,28]. Disruption of these finely regulated splicing programmes has been implicated in intellectual disability, epilepsy, autism spectrum disorder, movement disorders, and structural brain abnormalities.

Disease may also result from disruption of the splicing machinery itself. Small nuclear RNAs (snRNAs) are essential structural and catalytic components of the spliceosome and play central roles in splice-site recognition and spliceosome assembly. Recent studies have identified pathogenic variants in spliceosomal snRNA genes as an important cause of neurodevelopmental disease. De novo variants in *RNU4-2*, encoding U4 snRNA, cause ReNU syndrome and are associated with widespread disruption of splice-site usage. Similarly, dominant and recessive variants in *RNU2-2*, which encodes U2 snRNA and is required for branch-point recognition, cause severe neurodevelopmental disorders characterised by intellectual disability, epilepsy, hypotonia, microcephaly, and autistic features [9,10,29]. These disorders demonstrate that pathogenicity may arise not only from disruption of individual transcripts but also from impairment of the RNA-processing machinery responsible for transcriptome-wide splicing regulation.

### 2.3. Polyadenylation, Cleavage, and RNA Editing

Maturation of the mRNA 3′ end requires cleavage downstream of a polyadenylation signal (typically AAUAAA) followed by addition of a poly(A) tail. This process stabilises the transcript, promotes export, and supports translation [30]. Pathogenic variants in polyadenylation signals can impair transcript maturation without altering the coding sequence. A well-characterised example is the *HBB* polyadenylation signal mutation in β-thalassaemia, in which disruption of the AATAAA hexamer reduces β-globin mRNA output and causes haemolytic anaemia [31]. Alternative polyadenylation further regulates gene output by changing 3′ UTR length. Shortened 3′ UTRs may remove microRNA or RNA-binding protein sites and increase transcript stability or translational efficiency, whereas longer 3′ UTRs introduce additional regulatory elements. This mechanism is especially relevant in cancer, immune activation, and development.

### 2.4. RNA Editing

**Figure 3:**
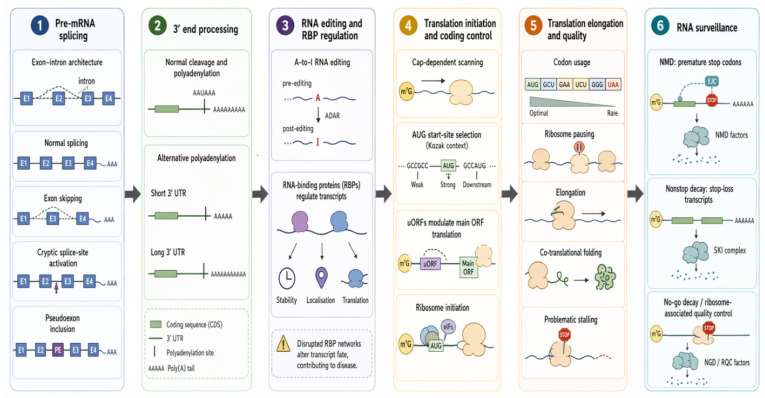
Major RNA-level Disease Mechanisms across Transcript Processing.

The most prominent mammalian RNA editing mechanism is adenosine-to-inosine (A-to-I) editing catalysed by ADAR1 and ADAR2. Inosine is interpreted as guanosine during translation and base pairing. A-to-I editing can alter coding potential, RNA structure, splice-site selection, RNA stability, localisation, and innate immune recognition [32]. In the nervous system, ADAR2-mediated recoding of the *GRIA2* transcript at the Q/R site of the GluA2 AMPA receptor subunit is nearly complete and essential: the edited arginine residue renders the receptor calcium-impermeable, and failure of this editing step causes lethal seizures in mice and has been implicated in amyotrophic lateral sclerosis in humans [33]. Loss-of-function variants in *ADAR1* impair editing of endogenous dsRNA, leading to accumulation of unedited dsRNA species that are sensed by MDA5 (encoded by *IFIH1*) as non-self, triggering a type I interferon response and the neuroinflammatory encephalopathy Aicardi–Goutières syndrome [12,32,33]. Gain-of-function variants in *IFIH1* produce a phenotypically overlapping condition by directly sensitising MDA5 to endogenous RNA [12]. Figure 3 illustrates major RNA-level disease mechanisms across transcript processing.

Splicing variants can cause exon skipping, cryptic splice-site use, or pseudoexon inclusion, altering transcript structure. Defects in 3′ end processing can disrupt transcript cleavage, impair canonical polyadenylation, or shift the balance of alternative polyadenylation site usage. RNA editing and RNA-binding proteins further modulate transcript identity, localisation, and translation. At the translational level, disruption of initiation, scanning, codon usage, elongation, or folding impairs protein synthesis. Aberrant transcripts are then subject to RNA surveillance (NMD, nonstop, no-go decay, and ribosome-associated quality control), ultimately converging on altered isoforms, abundance, and protein output.

### 2.5. mRNA Stability and RNA-Binding Proteins (RBPs)

Steady-state mRNA abundance reflects the balance between synthesis and degradation, modified by the 5′ cap, poly(A) tail, untranslated-region motifs, RNA structure, codon usage, RNA modifications, microRNA binding, and RNA-binding proteins. RNA-binding proteins coordinate splicing, export, localisation, stability, translation, storage, and decay; because they regulate large transcript networks, their disruption can produce broad, pleiotropic disease [13,14]. The nervous system is particularly vulnerable to RNA-binding protein dysfunction because neurons depend on long-distance RNA transport and local translation [15]. Fragile X syndrome illustrates dysregulated synaptic translation; spinal muscular atrophy reflects impaired ribonucleoprotein assembly and RNA processing [13,16]. HNRNPU-related disorder demonstrates neurodevelopmental consequences of disrupted RNA metabolism [29]; and FUS/TDP-43-associated neurodegeneration highlights the impact of altered RNA-binding protein localisation, aggregation, and stress-granule biology [34].

### 2.6. Surveillance Pathways

Nonsense-mediated mRNA decay (NMD) is a highly conserved RNA surveillance pathway that identifies and degrades transcripts containing premature termination codons (PTCs). PTCs commonly arise from nonsense variants, frameshift variants, aberrant splicing events, or pseudoexon inclusion. NMD is typically triggered when translation terminates more than approximately 50–55 nucleotides upstream of the final exon–exon junction, allowing exon-junction complexes to recruit the UPF1–UPF2–UPF3 surveillance machinery and initiate transcript degradation [35,36]. The clinical effect depends on whether the mutant transcript is degraded or escapes surveillance: degradation usually produces loss of function and haploinsufficiency, whereas escape may generate dominant-negative or toxic truncated proteins [35,36]. Hemizygous loss-of-function variants in UPF3B, a core NMD component, impair RNA surveillance, leading to dysregulation of neuronal transcript networks and causing intellectual disability, autism spectrum disorder, ADHD, and, less commonly, childhood-onset schizophrenia [37,38].

RNA surveillance extends beyond NMD. Nonstop decay targets transcripts lacking a functional termination codon. Stop-loss variants can convert a normal stop codon into a sense codon, causing ribosomes to translate into the 3′ untranslated region or poly(A) tail. Such transcripts may be unstable and degraded, leading to loss of mutant mRNA rather than stable C-terminal protein extension [39]. *FOXE3* and *KISS1R* stop-loss variants illustrate how this mechanism can influence disease expression [40].

No-go decay responds to stalled ribosomes resulting from strong RNA secondary structures, rare codon clusters, or defective elongation [41]. The stalled transcript is cleaved and degraded, while the incomplete nascent peptide is handled by ribosome-associated quality control (RQC). Defects in RQC components lead to the accumulation of incomplete translation products and are associated with neurological and neuromuscular phenotypes: biallelic loss-of-function variants in *NEMF*, which encodes a core RQC factor, cause a neuromuscular syndrome characterised by intellectual disability, axonal neuropathy, and cerebellar atrophy, underscoring the essential role of translational quality surveillance in post-mitotic tissues [42].

### 2.7. Non-Coding RNAs and Localisation Defects

MicroRNAs are short non-coding RNAs that regulate gene expression by guiding silencing complexes to target transcripts, most often through partially complementary sequences in the 3′ untranslated region. They repress translation and promote mRNA degradation. Because one microRNA can regulate many transcripts, and one transcript can be targeted by several microRNAs, microRNAs generate dense post-transcriptional regulatory networks [3]. Feingold syndrome type 2, caused by disruption of the *MIR17HG* locus encoding the miR-17-92 cluster, illustrates how loss of a microRNA cluster can produce a Mendelian developmental disorder [43]. Importantly, phenotypic overlap with MYCN-related Feingold syndrome shows that similar clinical features can arise from disruption of distinct regulatory layers: transcription factor dosage in one form and post-transcriptional microRNA regulation in another.

In cancer, microRNAs may act as oncogenes or tumour suppressors depending on their targets. Loss of a tumour-suppressive microRNA can increase oncogene expression, while overexpression of an oncogenic microRNA can suppress tumour suppressor transcripts [44]. MicroRNAs are also being explored as biomarkers and therapeutic targets, although delivery and target specificity remain challenging [4].

Long non-coding RNAs are transcripts longer than 200 nucleotides that lack major protein-coding capacity but regulate gene expression through diverse mechanisms. They may act locally in cis or distantly in trans. They can recruit chromatin regulators, modulate transcription, interact with RNA-binding proteins, regulate mRNA stability, or affect translation [5]. Clinically, long non-coding RNA disorders show that disease can arise even when the nearby coding gene is intact. Loss of a genomic region encoding a regulatory lncRNA may alter the dosage of an adjacent developmental gene. *CHASERR* provides a particularly instructive example: disruption of this lncRNA can increase *CHD2* expression in cis, leading to severe neurodevelopmental disease [45]. Long non-coding RNAs remain difficult to interpret clinically because many are tissue-specific, poorly conserved, and incompletely annotated. Whether their dysregulation contributes causally to neurodevelopmental or neurodegenerative disease remains an open question.

Circular RNAs are generated by back-splicing and form covalently closed RNA molecules. Their circular structure makes them relatively resistant to exonuclease-mediated degradation. They are enriched in the nervous system and are developmentally regulated. Some act as microRNA sponges, protein-binding scaffolds, or regulators of parental gene expression [5]. Although few monogenic disorders are currently attributed primarily to circular RNA dysfunction, circRNA biology intersects with several disease mechanisms. Splicing defects, RNA editing abnormalities, and RNA-binding protein dysfunction can alter circRNA biogenesis [5]. In the brain, particularly at synapses, circRNAs are enriched and developmentally regulated, suggesting potential roles in synaptic function. Whether their dysregulation contributes causally to neurodevelopmental or neurodegenerative disease remains an open question, and their clinical relevance will become clearer as transcriptomic methods mature [46].

Small nuclear RNAs are core structural and catalytic components of the spliceosome. De novo variants in RNU4-2 (U4 snRNA) cause ReNU syndrome, now recognised as one of the more frequent monogenic neurodevelopmental disorders, estimated to account for approximately 0.4% of cases in one large cohort, with patient RNA-sequencing demonstrating systematic disruption of 5′ splice-site usage. Pathogenic variants in RNU2-2 (U2 snRNA) cause a severe neurodevelopmental disorder with prominent epilepsy, with a large cohort study identifying 141 affected individuals across dominant and biallelic forms [9,10,29]. Small nucleolar RNAs guide rRNA modification; SNORD118-related leukoencephalopathy illustrates how disruption of a snoRNA can cause severe neurological disease through impaired RNA modification and ribosome function [8].

### 2.8. Dysregulation of Translation

#### 2.8.1. Cap-Dependent Initiation and Signalling Control

Translation initiation is often the rate-limiting step in protein synthesis. Disorders of initiation demonstrate how altered protein synthesis can cause disease despite an intact coding sequence. EIF2B-related vanishing white matter disease reflects defective regulation of translational stress adaptation [47]. EIF2S3-related MEHMO syndrome illustrates the developmental consequences of impaired initiation-complex function [48]. EIF4A3 is an RNA helicase of the DEAD-box family, active in both the exon junction complex and translation initiation and required to unwind secondary structure in the 5′ UTR during ribosomal scanning. EIF4A3-related Richieri–Costa–Pereira syndrome demonstrates that disruption of RNA helicase activity during translation initiation can selectively affect morphogenesis [49].

Similarly, hyperactivation of the upstream translational signalling pathway via *MTOR* gain-of-function, *PTEN* loss, or tuberous sclerosis complex mutations leads to excessive translation that alters brain development and drives cellular proliferation. The mTOR pathway links nutrient and growth signals to protein synthesis. Dysregulated mTOR signalling is implicated in neurodevelopmental disorders, epilepsy, overgrowth syndromes, tumour predisposition, and cancer [50]. Tuberous sclerosis complex, PTEN-related disorders, and *MTOR* gain-of-function disorders illustrate how excessive translational signalling can affect brain development, growth, and cellular proliferation [51].

#### 2.8.2. Integrated Stress Response

The integrated stress response (ISR) is a conserved translational control pathway that converges diverse stresses, such as ER stress, amino acid deprivation, viral infection, and oxidative stress, on phosphorylation of eIF2α, mediated by kinases including PERK/EIF2AK3, GCN2/EIF2AK4, PKR/EIF2AK2, and HRI/EIF2AK [52]. Variants affecting ISR regulators such as PPP1R15B, which participates in eIF2α dephosphorylation, have been associated with neurodevelopmental phenotypes, microcephaly, and growth impairment [53].

#### 2.8.3. Upstream Open Reading Frames and 5′ UTR Variants

The 5′ UTR is a major regulator of translation initiation and protein output. Upstream open reading frames (uORFs) are short coding sequences in the 5′ UTR that can reduce downstream protein production by causing ribosome dissociation or delayed reinitiation [54,55]. Hereditary thrombocythaemia due to THPO regulatory variants provides a classical example: loss of uORF-mediated repression increases thrombopoietin translation and drives excessive platelet production [56]. A genome-wide study of 5′ UTR variants identified numerous pathogenic variants altering protein translation through uORF creation, Kozak disruption, or RNA structural changes [54,57]. Splicing defects involving non-coding 5′ UTR exons represent a related under-recognised mechanism: the GJB1 c.-16-8_-14del deletion disrupts splicing at the interface between the untranslated leader and the first coding exon, causing X-linked Charcot–Marie–Tooth disease [58].

These variants are probably under-recognised in rare diseases. Standard exome analysis often deprioritises untranslated-region variants, and total mRNA abundance may appear normal when the defect lies primarily at the level of translation. Functional assessment may therefore require reporter assays, polysome profiling, ribosome profiling, or proteomic readouts rather than RNA sequencing alone.

Therapeutically, targeting uORF- and 5′ UTR-mediated translational defects is conceptually promising but remains at an early stage of clinical development. ASOs or small molecules could theoretically block inhibitory uORFs, remodel RNA structure, alter ribosome scanning, or restore access to the main coding sequence [59]. However, such approaches require direct evidence that the variant changes translation and that correction restores safe and appropriate protein dosage. These mechanisms illustrate a central principle of RNA-level disease: an otherwise intact coding sequence may still produce disease if the transcript is misinterpreted by the translational machinery.

#### 2.8.4. Codon Usage, Synonymous Variants, and Elongation Kinetics

Coding sequences contain regulatory information influencing splicing, RNA structure, transcript stability, codon optimality, and translation kinetics; consequently, synonymous variants may be pathogenic despite preserving the encoded protein sequence [60]. Codon optimality is a major determinant of mRNA stability [61], and abnormal ribosome pausing can impair co-translational folding, reduce protein stability, trigger mRNA decay, or activate ribosome-associated quality control [62,63]. Clinically important examples include *HBB* c.79G>A (haemoglobin E), which creates a cryptic splice site contributing to mild β-thalassaemia [64].

#### 2.8.5. Translation Termination and Readthrough

Termination efficiency depends on stop codon identity, surrounding nucleotide context, RNA structure, and competition between release factors and near-cognate tRNAs [65]. Under some circumstances, ribosomes read through stop codons and extend translation. Pathological readthrough can generate C-terminally extended proteins or destabilise transcripts through nonstop decay [66]. Therapeutically induced readthrough may partially rescue premature termination codons, but efficacy is highly dependent on variant context, transcript abundance, drug exposure, and protein tolerance.

#### 2.8.6. Elongation Factors and eIF5A Hypusination

Translation elongation requires delivery of aminoacyl-tRNAs, peptide bond formation, and ribosomal translocation. Elongation factors such as EEF1A2 and EEF2 are essential for efficient and accurate protein synthesis. Variants in elongation factors can cause neurodevelopmental disorders, epilepsy, intellectual disability, and movement phenotypes, highlighting the sensitivity of the nervous system to translational disruption [67].

A specialised elongation mechanism involves hypusination of eIF5A [68]. Hypusination is a unique post-translational modification required for eIF5A activity and depends on enzymes, including DHPS and DOHH [69,70]. Hypusinated eIF5A facilitates translation of difficult sequence contexts, particularly polyproline-rich motifs. Defects in this pathway impair elongation, promote ribosome stalling, and selectively disrupt protein networks important for neuronal development. Pathogenic variants affecting *DHPS*, *DOHH*, or *EIF5A* are associated with neurodevelopmental disorders characterised by developmental delay, intellectual disability, seizures, and microcephaly [68,69,71].

### 2.9. mRNA Localisation, Storage, and Local Translation

Disease can arise not only from the wrong transcript or wrong amount of transcript, but also from the right transcript being in the wrong place, stored incorrectly, or translated at the wrong time. mRNA localisation is mediated by cis-acting sequence elements in the 3′ UTR, recognised by RNA-binding proteins that transport ribonucleoprotein complexes along the cytoskeleton and maintain them in a silenced state until local signals trigger translation [11]. Disease can therefore arise even when transcript sequence and total abundance appear normal: the defect may involve RNA transport, storage, release from repression, or local translation.

Neurons depend on local translation for synaptic plasticity, axon guidance, dendritic remodelling, and circuit maturation [72]. Fragile X syndrome illustrates how loss of an RNA-binding translational regulator can dysregulate synaptic protein synthesis. In ALS and frontotemporal dementia, mislocalisation and aggregation of RNA-binding proteins such as FUS and TDP-43 disrupt RNA transport, stress-granule dynamics, and local RNA metabolism. SMN deficiency in spinal muscular atrophy may also affect axonal and synaptic RNA handling, contributing to selective motor-neuron vulnerability [73,74].

The clinical implication is that RNA-level disease cannot be inferred only from transcript abundance. A pathogenic variant may alter where an RNA goes, when it is translated, or whether it is trapped in abnormal ribonucleoprotein complexes. For precision therapeutics, this creates an additional challenge: correcting RNA sequence or abundance may be insufficient unless the restored transcript reaches the relevant cellular compartment and is translated at the appropriate time.

## 3. Diagnostic Approaches to Detect RNA-Level Defects

### 3.1. Short-Read Transcriptomics and Tissue-Aware Profiling

Short-read RNA sequencing (RNA-seq) can detect aberrant splicing, expression outliers, intron retention, exon skipping, pseudoexon inclusion, allele-specific expression, and fusion transcripts [22,23]. Its diagnostic utility has been demonstrated in cohorts where exome or genome sequencing was uninformative, increasing diagnostic yield substantially when combined with disease-relevant tissue analysis [22,23]. Diagnostic implementation requires tissue-aware reference datasets, standardised bioinformatic pipelines, and clear rules for incorporating transcriptomic evidence into variant classification frameworks [75,76].

### 3.2. Long-Read Transcriptomics and Structural Resolution

Long-read transcriptomics will add complementary value where short-read data are insufficient [77]. Full-length transcript sequencing can resolve complex isoforms, allele-specific splicing, repetitive regions, structural rearrangements, fusion transcripts, and transcript architectures that are difficult to reconstruct from short reads. This will be particularly important for repeat expansions, pseudogene-rich loci, complex splicing disorders, and isoform-specific disease mechanisms [78,79,80].

### 3.3. Functional Assays for Splicing, Translation, and Stability

Functional assays remain important for validating RNA-level defects and establishing pathogenicity beyond computational prediction. RT-PCR and minigene assays can confirm aberrant splicing events, while expression studies assess transcript abundance, allele-specific expression, and dosage effects. Variants may also disrupt translation without altering RNA levels. Ribosome profiling (Ribo-seq), polysome profiling, reporter assays, and proteomic analyses can identify defects in translation initiation, uORF utilisation, codon optimality, elongation kinetics, and protein output [81]. These approaches highlight the need to extend diagnostic interpretation beyond the genome and transcriptome to include the translatome and proteome, particularly for synonymous, untranslated-region, and other regulatory variants.

### 3.4. Allele-Specific Expression and RNA-Centric Variant Classification

Allele-specific expression analysis can detect monoallelic or imbalanced expression consistent with pathogenic variants even when the variant lies outside canonical splice sites or coding sequence [22,23]. Integration of transcriptomic evidence into clinical variant interpretation requires standardised pipelines and frameworks that explicitly assign pathogenicity weight to RNA-level consequences. An RNA-centric diagnostic approach should be considered whenever exome sequencing is negative or yields variants of uncertain significance, and when candidate variants are synonymous, intronic, untranslated, splice-regulatory, stop-loss, repeat-associated, or located in non-coding RNA genes.

## 4. Therapeutic Strategies Matched to Specific RNA Mechanisms

RNA-level disease mechanisms are therapeutically attractive because RNA is sequence-specific, dynamically regulated, and accessible to programmable intervention. Unlike permanent DNA editing, most RNA-targeted approaches act reversibly and can be titrated in dose and duration. Unlike conventional protein-targeted drugs, RNA therapeutics act upstream of protein production by modifying transcript abundance, structure, splicing, translation, or surveillance, making them particularly suited to disorders caused by haploinsufficiency, toxic gain of function, aberrant splicing, pseudoexon inclusion, repeat-associated RNA toxicity, untranslated-region dysfunction, and dysregulated translation.

Table 1 summarises the major classes of RNA-targeted therapeutics, their mechanistic applications, representative disease examples, key limitations, and supporting references.

### 4.1. Antisense Oligonucleotides (ASOs): Programmable Correction of Transcript Fate

ASOs are short, synthetic, single-stranded, chemically modified nucleic acids that bind complementary RNA sequences through Watson–Crick base pairing [95,97]. Steric-blocking ASOs bind RNA motifs without inducing transcript degradation, redirecting RNA processing by masking splice sites, splicing silencers or enhancers, or pathogenic RNA–protein interaction sites [95]. The clinical paradigm is spinal muscular atrophy: nusinersen binds the intronic splicing silencer ISS-N1 downstream of SMN2 exon 7, preventing repressor binding, promoting exon 7 inclusion, and increasing full-length SMN protein expression [98]. In Duchenne muscular dystrophy, exon-skipping ASOs restore the dystrophin reading frame, producing a shorter in-frame dystrophin resembling the milder Becker phenotype [83,99]. In pseudoexon disorders, steric-blocking ASOs can mask cryptic splice sites created by deep intronic variants, restoring normal splicing [84].

RNase H1-dependent gapmer ASOs are designed to reduce target RNA rather than remodel it. After hybridisation, the DNA gap of the gapmer forms an RNA–DNA duplex recognised by RNase H1, leading to cleavage of the RNA strand and reduction in transcript abundance [97]. This mechanism is best suited to diseases caused by toxic RNA, toxic protein production, dominant gain-of-function alleles, or pathogenic overexpression. Tofersen reduces SOD1 mRNA in SOD1-associated ALS [20]; similar transcript-depletion approaches are being explored in repeat expansion disorders [21]. Limitations of ASO therapy include tissue delivery constraints, repeated dosing requirements, off-target hybridisation, immune activation, renal or hepatic toxicity, and thrombocytopenia [100,101,102,103].

### 4.2. Splice-Modifying Small Molecules

Small molecules can modulate splicing by binding RNA secondary structures, spliceosomal components, or RNA–protein complexes to influence splice-site selection [87,104]. Their principal advantage is oral bioavailability and systemic tissue distribution. Risdiplam promotes SMN2 exon 7 inclusion by strengthening U1 snRNP recognition of the exon 7–intron 7 5′ splice site, shifting processing toward full-length *SMN2* mRNA [24,87]. Risdiplam is relatively *SMN2*-selective, but the FDA label notes that off-target splicing effects on *FOXM1* and *MADD* occur at therapeutic concentrations [87]. For rare monogenic disorders, splice-modifying small molecules are most attractive when a recurrent splicing defect can be targeted across many patients.

### 4.3. RNA Interference (siRNA) and Transcript Silencing

siRNAs exploit the endogenous RNA interference pathway: the antisense guide strand is incorporated into RISC, which directs sequence-specific cleavage of complementary mRNA, reducing target transcript abundance [105,106]. Clinical success has been greatest in liver-directed disorders, where hepatocyte delivery is achieved using lipid nanoparticles or GalNAc-conjugated platforms. Patisiran and vutrisiran suppress hepatic transthyretin production in hereditary transthyretin amyloidosis [17]; givosiran reduces *ALAS1* expression in acute hepatic porphyria [18,19]; lumasiran lowers glycolate oxidase expression in primary hyperoxaluria type 1 [86]; tofersen targets *SOD1* in familial ALS [20,21]; inclisiran reduces PCSK9 expression to lower LDL cholesterol [85,107,108]. This mechanism is not suitable for haploinsufficient disorders where further reduction in gene expression would be harmful. It is ideal for toxic gain-of-function variants or dominant-negative disorders where a mutant transcript produces a deleterious protein or sequesters vital cellular factors, in which sequence-specific transcript knockdown is required.

### 4.4. Synthetic mRNA Replacement Therapy

In genetic states characterized by severe haploinsufficiency or complete loss of function, synthetic, translation-competent mRNA replacement can deliver mature messages directly into the cytoplasm. This completely bypasses genomic promoter defects, splicing mutations, and abnormal upstream RNA processing. Optimized using modified nucleosides, modified poly(A) tails, and lipid nanoparticle (LNP) formulations to maximize stability and prevent innate immune sensing, mRNA replacement is highly effective for systemic metabolic enzyme deficiencies or secreted factors where transient, pulsatile protein production is therapeutic. mRNA replacement therapy delivers a synthetic, translation-competent transcript encoding the therapeutic protein. By supplying the mature message directly, it bypasses defects in the endogenous gene, promoter regulation, splicing, and upstream RNA processing. This makes it conceptually attractive for loss-of-function disorders, especially when transient or repeated production of a secreted protein, enzyme, or circulating factor can provide systemic benefit [109,110]. The success of mRNA vaccine platforms has established that synthetic mRNA can be manufactured and delivered at scale. For monogenic diseases; however, the therapeutic requirements are more stringent: protein expression may need to be sustained, tissue-specific, repeatedly dosed, and quantitatively controlled.

### 4.5. Programmable RNA Editing and Readthrough Modulators

Programmable RNA editing leverages site-directed correction at the transcript level without modifying the genomic architecture. By employing engineered guide RNAs to recruit endogenous ADAR enzymes, precise A-to-I (functionally A-to-G) conversions can fix point mutations or eliminate premature stop codons. Guide RNAs recruit ADAR activity to specific transcript positions, enabling site-directed correction of selected RNA variants [12,90]. The investigational RNA-editing oligonucleotide WVE-006 targets the SERPINA1 Z allele in alpha-1 antitrypsin deficiency [111]. Preclinical demonstrations include LEAPER 2.0 for Hurler syndrome (IDUA nonsense mutations in humanised mouse and non-human primate models) [97], *MECP2* editing in Rett syndrome neuronal models [96], and CFTR transcript correction [98]. RNA editing remains an early-stage platform; challenges include editing efficiency, off-target recoding, delivery, durability, and immune activation [11].

Readthrough therapy attempts to restore protein production by allowing ribosomal decoding of a premature stop codon as a sense codon [112]. Aminoglycosides such as gentamicin provided early proof of concept in DMD [113] and cystic fibrosis [114], but chronic toxicity limited clinical use. Ataluren was developed as an orally administered readthrough agent for DMD; its regulatory history illustrates the difficulty of translating readthrough biology into consistent clinical benefit [91]. ELX-02, a synthetic aminoglycoside derivative with reduced toxicity, has shown preclinical CFTR rescue and entered early clinical evaluation [115]. Efficacy is highly context-dependent: the stop codon identity, surrounding nucleotide context, transcript stability, NMD status, and protein tolerance for an amino acid insertion at the premature stop must all be favourable [92,112].

### 4.6. Modulating NMD and RNA Surveillance

NMD is therapeutically double-edged. Transient inhibition may be useful when stabilising a PTC-containing transcript increases readthrough substrate or permits production of a protein with residual activity: NMD inhibition in W1282X-CFTR can increase transcript abundance for rescue by modulators [92]; similar logic has been explored in DMD [116,117]. Conversely, when the abnormal RNA or protein is toxic, therapeutic strategies should favour transcript depletion. In cancer, NMD inhibition may increase mutant transcript expression and neoantigen availability, with preclinical studies suggesting synergy with immune checkpoint blockade [116]. Broad NMD inhibition risks stabilising harmful transcripts and disrupting normal cellular homeostasis; clinically viable approaches will likely need to be transient, tissue-targeted, or combined with complementary RNA-targeted strategies [92,93,116].

### 4.7. Targeting Non-Coding RNAs and RNA Regulatory Networks

Non-coding RNAs can be therapeutic targets and therapeutic agents. Anti-miRNA oligonucleotides inhibit pathogenic microRNAs; miRNA mimics aim to restore deficient regulatory activity. Miravirsen (anti-miR-122) produced dose-dependent and prolonged reductions in HCV RNA in a Phase 2a trial because miR-122 is required by HCV for RNA stabilisation [118]. MRX34, a liposomal miR-34a mimic, was terminated after immune-related serious adverse events in a Phase 1 trial, illustrating the risks of non-coding RNA network-level modulation [44]. LncRNA-directed therapy using RNase H1-dependent gapmer ASOs has shown preclinical efficacy in depleting MALAT1 in selected cancer models [119], but challenges include nuclear localisation and context-dependent biological effects. Circular RNA platforms are being explored for long-acting protein expression and vaccine applications [120].

### 4.8. RNA-Targeting Small Molecules

RNA-targeting small molecules recognise RNA structure or RNA–protein complexes and alter RNA behaviour pharmacologically, without relying on long sequence-complementary hybridisation [109]. In myotonic dystrophy type 1 and 2, small molecules that bind expanded r(CUG) and r(CCUG) repeats have been shown experimentally to reverse molecular defects and stimulate degradation of pathogenic repeat-containing RNA through RNA decay pathways [121,122]. The attraction is oral delivery and conventional drug-like manufacturing; the main challenge is selectivity, requiring high-resolution RNA structural biology and transcriptome-wide binding assays [123].

### 4.9. Personalised RNA Therapeutics

Personalised RNA therapeutics extend mechanism-matched treatment to ultra-rare or private variants, exploiting the fact that oligonucleotide therapies can be designed directly from sequence information [124]. Milasen, a patient-customised splice-modulating ASO for CLN7-related Batten disease, established a practical template for N-of-1 oligonucleotide therapy [124]. Jacifusen/ION363, developed for FUS-ALS, illustrates how personalised ASO therapy can be used for transcript lowering rather than splice correction [125]. The n-Lorem Foundation systematises personalised ASO development for ‘nano-rare’ patients [126]. These approaches challenge standard regulatory and development models, requiring molecular confirmation of the defect, evidence of correction in patient-derived models, proportionate safety testing, and clear clinical endpoints [127].

### 4.10. Matching Therapeutic Strategy to RNA Mechanism

**Figure 4:**
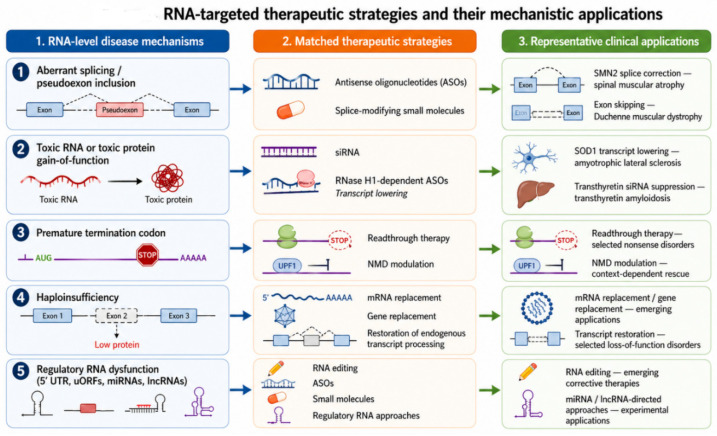
RNA-targeted Therapeutic Strategies and their Mechanistic Applications. Splicing defects and pseudoexon inclusion are corrected by ASOs or splice-modulating small molecules; toxic gain-of-function transcripts are reduced by siRNA or RNase H1-dependent ASOs; premature termination codons are targeted by readthrough or NMD-modulating therapies; haploinsufficiency may require mRNA or gene replacement. Emerging approaches address UTR, uORF, miRNA, and lncRNA defects through RNA editing, ASOs, small molecules, and regulatory RNA modulation. Representative examples include SMN2 splice correction [98], DMD exon skipping [83], SOD1 knockdown [20], and TTR silencing [17].

RNA therapeutics should be selected according to the dominant molecular consequence of the variant, not the gene name or variant class alone. A nonsense variant is not automatically a readthrough candidate: suitability depends on transcript abundance, NMD sensitivity, stop-codon context, and whether the restored protein would be functional and safe. A dominant disorder is not automatically suitable for silencing: transcript depletion is appropriate for toxic gain of function but may worsen haploinsufficiency. A splice variant is not automatically correctable unless the restored transcript is stable, functional, and deliverable in the relevant tissue. Figure 4 summarises the major classes of RNA-targeted therapeutics and their mechanistic applications.

## 5. Clinical Maturity and Translational Limitations

Although RNA therapeutics have progressed rapidly from conceptual frameworks to clinical implementation, the maturity of individual platforms varies substantially. Splice-modulating ASOs and siRNA therapies represent the most clinically validated RNA-targeted modalities, with multiple regulatory approvals and demonstrated efficacy in defined genetic disorders. In contrast, RNA editing, NMD modulation, many non-coding RNA therapeutics, and personalised N-of-1 interventions remain investigational and are supported primarily by preclinical studies or early clinical experience [44,90,92,109]. The current clinical status, representative examples, and major limitations of these therapeutic classes are summarised in Table 2.

### 5.1. The Delivery Barrier and Tissue Selectivity

Efficient and tissue-specific delivery remains the principal technical limitation of RNA therapeutics [100,101,102,103,106]. Clinical success has been greatest in the liver, where hepatocyte uptake can be achieved through lipid nanoparticles and N-acetylgalactosamine (GalNAc) conjugation, enabling effective delivery of siRNA and other oligonucleotide-based therapies [121,123]. Similarly, intrathecal administration has enabled treatment of neuromuscular and central nervous system disorders by bypassing the blood–brain barrier, as demonstrated by therapies such as nusinersen [128] and tofersen [20,103]. In contrast, achieving efficient delivery to widespread brain parenchyma, skeletal muscle, cardiac muscle, lung, kidney, and retina remains considerably more challenging. These tissues often require high systemic doses, increasing the risk of off-target effects and toxicity. Consequently, considerable effort is focused on developing next-generation delivery platforms, including engineered lipid nanoparticles, viral vectors, tissue-targeted ligands, polymer conjugates, and exosome-based delivery systems, with the aim of expanding the range of clinically accessible tissues [100,102]. The clinical success of siRNA therapy has been greatest in liver-directed disorders, because hepatocyte delivery can be achieved efficiently using lipid nanoparticles or GalNAc-conjugated platforms. This makes siRNA particularly suitable for diseases in which the liver produces a pathogenic protein, toxic metabolite, or disease-modifying circulating factor [105,106].

### 5.2. Durability and Repeated Dosing

Most RNA therapies require repeated administration because they modify RNA rather than permanently correcting the underlying genomic variant. ASOs and siRNAs have intermediate half-lives in tissue, ranging from weeks to months depending on chemistry and tissue, but a persistent pharmacological effect requires re-dosing [85,86]. This has implications for patient adherence, manufacturing costs, and long-term safety monitoring. mRNA replacement requires repeated dosing for sustained protein expression, with each administration carrying a risk of innate immune activation [94,95].

### 5.3. Off-Target Hybridization, Toxicity, and Dosing Liabilities

Potential safety concerns include off-target hybridisation (leading to unintended transcript suppression or splicing changes), innate immune activation, renal or hepatic toxicity, thrombocytopenia (for certain ASO chemistries), and toxicity associated with delivery systems [79,80,81,82]. Long-term safety data remain limited for most modalities. The termination of MRX34 due to immune-related serious adverse events highlights the risks of broad network-level modulation by non-coding RNA therapeutics [38]. Off-target splicing effects of risdiplam, including alterations in FOXM1 and MADD splicing, are noted in clinical prescribing information [83].

### 5.4. The Phenotypic Gap: Molecular Rescue vs. Clinical Outcome

A major translational limitation is the prominent gap between successful molecular rescue and meaningful clinical benefit. Many emerging RNA therapeutic strategies demonstrate highly elegant proofs of mechanism in cell-culture or mouse models, such as clearing toxic RNA foci or increasing local transcript abundance, without translating into measurable clinical outcomes in human trials.

Clinical efficacy is strongly influenced by developmental timing and the extent of irreversible pathology present at treatment initiation. Experience in spinal muscular atrophy has demonstrated that treatment with nusinersen, risdiplam, or gene replacement therapy produces substantially greater benefit when initiated presymptomatically or early in disease progression, before significant motor neuron loss has occurred [129,130]. These findings highlight that successful correction of an RNA defect may not fully reverse established developmental or degenerative pathology.

Furthermore, successful target engagement does not necessarily translate into meaningful clinical improvement. In Huntington’s disease, antisense-mediated lowering of mutant huntingtin demonstrated biological activity and reduction in the target protein, yet clinical outcomes did not meet expectations in later-stage trials, illustrating the complexity of linking molecular correction to patient benefit [131].

Similar challenges have been observed in amyotrophic lateral sclerosis, where RNA-targeted therapies have demonstrated biomarker effects and evidence of target engagement, while the magnitude and timing of clinical benefit continue to be actively evaluated [132]. Such studies emphasise the importance of distinguishing molecular efficacy from clinically meaningful outcomes.

### 5.5. Regulatory Status and Approved Therapies

Several RNA-based therapies have received regulatory approval, demonstrating clinical feasibility. Nusinersen (SMA, 2016), eteplirsen and related exon-skipping ASOs (DMD), tofersen (SOD1-ALS), and multiple siRNA therapeutics (patisiran, vutrisiran, givosiran, lumasiran, inclisiran) are among the approved agents [23,77,83,87,88,89,90,91,92,93]. The regulatory landscape continues to evolve, particularly for newer modalities such as RNA editing, NMD modulation, and N-of-1 personalised interventions, where conventional trial designs and toxicology frameworks require adaptation [115].

## 6. Future Directions

The next phase of genomic medicine will require a transition from variant-centred interpretation to mechanism-guided diagnosis and therapy. An RNA-centric framework links three key steps: identifying transcript-level abnormalities, defining the dominant pathogenic RNA mechanism, and selecting an appropriate therapeutic intervention. This approach is particularly relevant when exome sequencing is non-diagnostic or when candidate variants are synonymous, intronic, untranslated, splice-regulatory, repeat-associated, or located within non-coding RNA genes.

### 6.1. Artificial Intelligence and Predictive Modelling

Artificial intelligence and machine learning approaches are increasingly being applied to predict splice-site disruption, cryptic splice activation, RNA secondary structure, RNA-binding protein interactions, upstream open reading frame function, translation efficiency, and therapeutic oligonucleotide performance [133]. These tools have the potential to accelerate variant prioritisation and therapeutic design, particularly within the non-coding genome. However, computational predictions should be regarded as hypothesis-generating and require validation through transcriptomic, functional, and clinical evidence before informing diagnostic or therapeutic decisions.

### 6.2. Multi-Omic Integration and N-of-1 Regulatory Architectures

Future diagnostic workflows are likely to integrate genomic, transcriptomic, translatomic, proteomic, and phenotypic data to improve variant interpretation and disease mechanism discovery. Broader implementation of RNA sequencing, standardised analytical pipelines, tissue-specific reference datasets, and improved frameworks for incorporating transcriptomic evidence into variant classification will be essential [23,94,134]. Long-read transcriptomics will provide complementary insights into complex isoforms, allele-specific splicing, repetitive regions, and transcript architectures that are difficult to resolve using short-read approaches [77]. Functional assays, including ribosome profiling, polysome profiling, reporter systems, and quantitative proteomics, may further improve interpretation of variants whose primary effects occur at the level of translation rather than transcript abundance [81].

### 6.3. Personalised RNA Therapeutics and Regulatory Challenges

Advances in programmable RNA technologies are creating opportunities for highly personalised therapies, including patient-specific antisense oligonucleotides and genotype-directed RNA interventions. The development of milasen for CLN7-associated Batten disease established a proof-of-principle framework for personalised RNA therapy, demonstrating how identification of an aberrant splicing event can be translated into a custom therapeutic intervention [94,124]. More recently, investigational therapies such as jacifusen for FUS-associated amyotrophic lateral sclerosis have illustrated the broader potential of genotype-specific RNA targeting [96,125].

These advances challenge traditional regulatory and clinical trial paradigms. For ultra-rare or unique pathogenic variants, conventional large-scale efficacy studies may be impractical. Future implementation will require regulatory frameworks that balance rapid therapeutic development with rigorous evaluation of safety, manufacturing quality, long-term monitoring, and equitable access. Addressing these challenges will be essential for the broader adoption of RNA-guided precision medicine.

## 7. Conclusions

**Figure 5:**
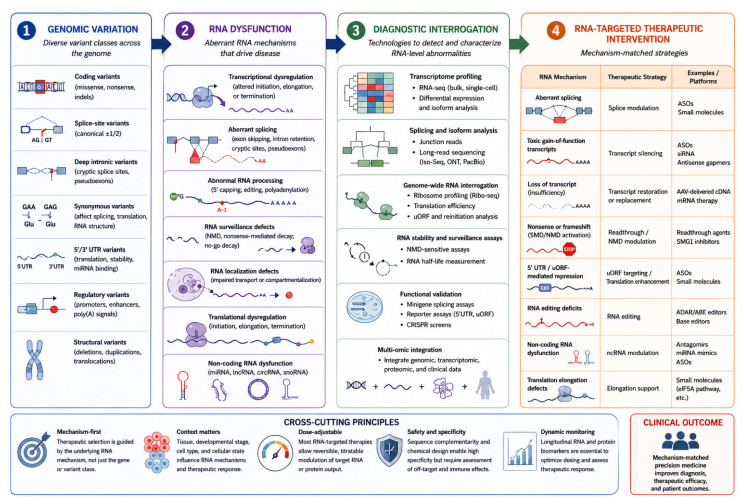
Mechanism-matched framework for RNA-guided precision medicine.

RNA-level mechanisms play a central role in human disease and provide a versatile platform for diagnostic and therapeutic innovation. By linking molecular mechanisms to targeted interventions, RNA-based approaches offer significant potential for precision medicine. However, substantial challenges remain in achieving consistent clinical translation. Continued integration of mechanistic insights, diagnostic tools, and therapeutic development will be critical to fully realise the promise of RNA-targeted strategies. The integrated clinical workflow for mechanism-based therapeutic selection, from variant detection through RNA consequence to matched intervention, is summarised in Figure 5.

Genetic variants produce disease through a range of RNA-level mechanisms that may not be predicted by variant class alone. Identification of the dominant RNA abnormality through transcriptomic and functional interrogation enables selection of therapeutic strategies aligned to the underlying mechanism, including splice correction, transcript suppression, transcript restoration, translational rescue, RNA editing, and non-coding RNA modulation. The framework illustrates the progression from genotype to RNA dysfunction, diagnostic assessment, and mechanism-matched therapeutic intervention.

The integrated clinical workflow for mechanism-based therapeutic selection, from variant detection through RNA consequence to matched intervention, is summarised in Figure 5.

**Figure 6:**
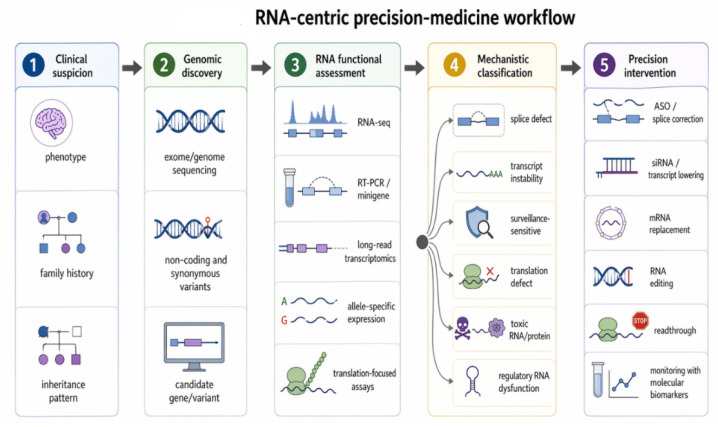
RNA-centric Precision-Medicine Workflow.

The complete framework, from variant class through RNA-level mechanism, diagnostic assay, and therapeutic strategy, is summarised in Figure 6, which serves as a visual reference for mechanism-matched clinical decision-making.

An RNA-centric precision-medicine workflow connects clinical phenotype, genomic sequencing, RNA functional testing, mechanistic classification, and targeted therapy. RNA assays define variant effect beyond DNA, distinguishing splicing, instability, translational, toxic, and regulatory defects. These mechanisms directly map to interventions including ASOs, siRNA, mRNA replacement, RNA editing, and readthrough therapy, enabling mechanism-guided diagnosis and treatment.

## Figures and Tables

**Table 1:** RNA-targeted therapeutic strategies, mechanisms, examples, and limitations.

Therapeutic Strategy	Primary Molecular Action	Best-Suited RNA-Level Disease Mechanism	Representative Examples or Clinical Settings	Precision-Medicine Rationale	Principal Limitations	References
**Steric-blocking antisense oligonucleotides**	Bind pre-mRNA or mRNA without inducing degradation; alter splice-site recognition or block regulatory motifs	Exon skipping, pseudoexon inclusion, exon exclusion, aberrant splice-site activation	Nusinersen for SMA; exon-skipping approaches in Duchenne muscular dystrophy; personalised ASOs for rare pseudoexon disorders	Directly corrects transcript architecture while preserving endogenous gene regulation	Tissue delivery, repeated dosing, intrathecal administration for CNS disease, off-target hybridisation, variant specificity	[82,83,84]
**RNase H1-dependent gapmer ASOs**	Recruit RNase H1 to degrade target RNA	Toxic gain-of-function transcripts, dominant alleles, expanded repeat transcripts, pathogenic overexpression	Tofersen for SOD1-ALS; ASO approaches for Huntington disease and repeat expansion disorders	Reduces production of toxic RNA or toxic protein at transcript level	Allele specificity may be required; excessive knockdown may be harmful; CNS delivery remains challenging	[20]
**siRNA therapeutics**	Use RNA-induced silencing complex to degrade complementary mRNA	Hepatic gain-of-function disease, toxic protein production, metabolic pathway overactivity	Patisiran and vutrisiran for transthyretin amyloidosis; givosiran for acute hepatic porphyria; lumasiran for primary hyperoxaluria; inclisiran for LDL-cholesterol reduction	Durable and potent transcript silencing, especially in liver-targeted disease	Delivery beyond liver is less mature; not ideal for haploinsufficiency; potential on-target toxicity if normal transcript is required	[17,19,85,86]
**Splice-modifying small molecules**	Bind RNA or spliceosomal components to alter exon inclusion	Splicing defects where transcript correction can restore functional protein	Risdiplam for SMA; investigational splice modulators in neurological, oncological, and rare-disease settings	Orally deliverable alternative to ASOs in selected disorders	Lower sequence specificity than ASOs; systemic exposure; risk of off-target splicing changes	[87]
**mRNA replacement therapy**	Delivers synthetic mRNA encoding a therapeutic protein	Loss-of-function or haploinsufficient disease where protein replacement is sufficient	mRNA vaccines; investigational mRNA enzyme/protein replacement therapies	Bypasses defective endogenous gene and transcript processing; transient and titratable	Repeated dosing, innate immune activation, delivery constraints, protein dosage control, tissue targeting	[88,89]
**RNA editing**	Rewrites RNA sequence, most commonly via ADAR-mediated A-to-I editing	Pathogenic single-nucleotide transcript changes, selected splice or coding defects	Programmable ADAR-recruiting systems in development	Potentially reversible correction without permanent genome editing	Efficiency, specificity, delivery, off-target editing, immunogenicity, limited clinical maturity	[90]
**Readthrough therapy**	Promotes ribosomal readthrough of premature termination codons	Nonsense variants where full-length or near-full-length protein would be functional	Aminoglycoside derivatives; ataluren and related compounds investigated in DMD, cystic fibrosis, and other nonsense-mediated disorders	Targets a specific class of loss-of-function variants	Highly context-dependent; not useful if transcript is degraded by NMD; risk of global termination errors; variable clinical efficacy	[91,92]
**NMD modulation**	Alters degradation of premature-termination-codon-containing transcripts	Disorders where NMD removes transcripts that could encode partially functional protein, or where enhanced decay could reduce toxic products	Mostly experimental; potential combination with readthrough or ASO strategies	Connects variant interpretation directly to transcript fate	NMD regulates many normal transcripts; broad inhibition may be toxic; requires precise prediction of protein consequence	[93]
**Anti-miRNA and miRNA replacement therapy**	Inhibits pathogenic miRNAs or restores deficient miRNA activity	miRNA-mediated over-repression or loss of post-transcriptional regulation	Investigational cancer, cardiovascular, fibrotic, and inflammatory disease programmes	Modulates regulatory networks rather than a single protein	Pleiotropy, delivery, immune activation, narrow therapeutic window	[44,94,95]
**RNA-targeted small molecules**	Bind structured RNA elements or RNA-protein interfaces	Repeat expansion RNA toxicity, structured UTR-mediated translation, pathogenic RNA-protein interactions	Small molecules targeting repeat RNAs or splice-regulatory RNA structures in development	Oral drug-like approach to RNA biology	RNA structural plasticity, target selectivity, off-target binding, early-stage validation	
**Gene therapy affecting RNA output**	Delivers a functional gene copy or modifies expression of a transcript-relevant gene	Severe loss-of-function disease where durable replacement is preferable to repeated RNA dosing	AAV-based gene replacement for selected monogenic disorders; SMA gene-replacement approaches	Provides sustained expression and may reduce need for repeated RNA therapy	Not strictly RNA therapy; irreversible or long-lived exposure; immune issues; vector packaging limits; dose-related toxicity	[96]

**Table 2:** Clinical maturity of RNA-targeted therapeutic modalities.

Therapeutic Modality	Clinical Maturity	Representative Examples	Major Limitations	References
Splice-modulating ASOs	Approved (regulatory)	Nusinersen (SMA), eteplirsen (DMD exon skipping)	Repeated dosing; tissue delivery; intrathecal CNS administration	[23,75,83,84,122]
siRNA therapeutics	Approved (multiple)	Patisiran, vutrisiran (transthyretin amyloidosis); givosiran (AHP); lumasiran (PH1); inclisiran (hypercholesterolaemia)	Primarily liver-targeted; extrahepatic delivery limited	[87,88,89,90,91,92,93]
mRNA replacement	Early clinical/investigational	mRNA vaccines (COVID-19, proof-of-concept); mRNA for PKU, PH1 (preclinical/early)	Durability; repeated dosing; immunogenicity; delivery	[94,95,123,124]
Readthrough therapy	Limited and variable clinical success	Ataluren (DMD, regulatory history); ELX-02 (CF); gentamicin (early proof-of-concept)	Highly context-dependent; variable efficacy; NMD must not eliminate substrate	[100,101,102,104,125]
RNA editing (ADAR-based)	Preclinical/early clinical	WVE-006 (AATD, clinical); LEAPER 2.0 (NHP); Rett/Hurler models	Editing efficiency; delivery; off-target recoding; limited clinical maturity	[11,96,97,98,99]
NMD modulation	Experimental	W1282X-CFTR studies; DMD/NMD preclinical; cancer neoantigen preclinical	Global pathway effects; broad inhibition toxic; mainly experimental	[24,105,126]
Non-coding RNA therapeutics	Early clinical/experimental	Miravirsen (anti-miR-122, HCV, Phase 2a); MRX34 (miR-34a mimic, terminated)	Network complexity; immune activation; MRX34 terminated due to SAEs	[38,106]
Personalised ASOs (N-of-1)	N-of-1 clinical implementation	Milasen (CLN7 pseudoexon, splice correction); jacifusen/ION363 (FUS-ALS, transcript lowering); n-Lorem Foundation programme	Scalability; regulatory frameworks; manufacturing; equitable access	[112,113,114,115]

## Data Availability

No new data were generated or analysed in this study. Data sharing does not apply to this article.
